# Metabolome and transcriptome analyses provide insights into the podophyllotoxin content difference in different *Sinopodophyllum hexandrum* provenances

**DOI:** 10.3389/fpls.2025.1722118

**Published:** 2026-01-05

**Authors:** Wei Liu, Sensen Chen, Xiaoqiu Yuan, Zheng Zhang, Kai Duan, Longwei Li

**Affiliations:** 1 College of Agriculture, Henan University of Science and Technology, Luoyang, China; 2 Luoyang Forestry Ecological Construction and Development Center, Luoyang, China

**Keywords:** candidate genes, metabolome, podophyllotoxin, *Sinopodophyllum hexandrum*, transcriptome

## Abstract

*Sinopodophyllum hexandrum* is a perennial herb medicinal plant and mainly distributes in high-altitude areas. Its rhizome is a primary material source for anticancer active ingredient podophyllotoxin (PTOX) production. The PTOX content was significantly different in rhizomes of different provenances, but this formation reason remains unclear. In this study, *S. hexandrum* provenances of ShaanXi (SX), GanSu (GS) and Tibet (XZ) were collected from the resource nursery of *S. hexandrum*. Through the combined analysis of non-targeted metabolomics and transcriptomics, candidate genes, transcription factors, and transporters significantly related to the PTOX content difference were screened to reveal the formation reason of the difference in PTOX content from different provenances. The results showed that deoxypodophyllotoxin synthase (2-ODD), secoisolariciresinol dehydrogenase (SDH) and coumarate 3-hydroxylase (C3H) were essential genes that lead to the PTOX content differences in *S. hexandrum* from different provenances, WRKY and AP2/ERF-ERF were considered to be key transcription factors, and ABCE1 and ABCC2 were the primary transporters. The results can provide a new perspective and excellent genes for revealing the cause of the different PTOX contents in *S. hexandrum* from different provenances.

## Introduction

Podophyllotoxin (PTOX) is an aryl tetrahydronaphthalene lactone lignan and has significant biological activities, including anti-tumor, anti-virus, hypolipidemic, and immunosuppression effects (Jin et al., 2023; Kumari et al., 2022; Motyka et al., 2023). PTOX generates anti-tumor effect mainly by inhibiting the polymerization of tubulin and DNA topoisomerase II (DNA TII) from causing cell proliferation arrest and inhibiting the mitotic spindle formation (Wen et al., 2019). Previous reports demonstrated that PTOX and its semi-synthetic glycoside derivatives such as etoposide, teniposide, and etoposide are highly active anti-tumor drugs, which can be widely used in clinical practices for treating small cell lung cancer, leukemia, and testicular cancer (Guo et al., 2023; Guo and Jiang, 2021; Zhao et al., 2021). PTOX has important medicinal value and high market demand. Currently, PTOX has been produced by artificial cultivation (Sharma et al., 2022), chemical synthesis (Xiao et al., 2018), and biotechnology intervention (Kitaeva et al., 2023).

*Sinopodophyllum hexandrum* is a perennial herb medicinal plant in *Sinopodophyllum* genus in Berberidaceae family. *S. hexandrum* is mainly distributed in China, India and Nepal. In China, it widely grows in high-altitude areas such as ShaanXi (SX), Tibet (XZ) and Gansu (GS) provinces (Liu et al., 2021). Its rhizome is a primary source for PTOX (Cao et al., 2021). The genetic diversity of *S. hexandrum* is rich, and the content of PTOX varies from different populations (Liu et al., 2014; Xu, 2023). There are many studies on PTOX in *S. hexandrum*, mainly focusing on the determination of PTOX content (Feng et al., 2020), fingerprint analysis (Liu et al., 2021), and biosynthetic pathway analysis (Danaeipour et al., 2023). At present, the biosynthesis pathway of PTOX has been preliminarily revealed. Firstly, the precursor compound coniferyl alcohol was synthesized from phenylpropanoid by the phenylpropanoid pathway. Coniferyl alcohol was used as a co-synthesis precursor of PTOX and its derivatives to synthesize pinoresinol under the action of the dirigent protein (DIR) protein. Secondly, pinoresinol was catalyzed to form matairesinol by pinoresinol-lariciresinol reductase (PLR) and secoisolariciresinol dehydrogenase (SDH) enzymes. Matairesinol is converted into deoxypodophyllotoxin, and deoxypodophyllotoxin finally forms PTOX (Danaeipour et al., 2023). Lignans mostly exist in plants in the form of glycosylation, and the enzymes catalyzing for glycosylation remain unknown at present (Meng et al., 2021).

Liu et al. (2025) found *ShOMT3* was an important enzyme gene involved in the downstream synthesis of PTOX from pluviatolide to deoxypodophyllotoxin in *S. hexandrum*. The role of this enzyme gene in the PTOX biosynthesis pathway has also been characterized in other reports. Lau and Sattely (2015) conducted a transcriptome analysis of *Podophyllum hexandrum* (synonym for *S. hexandrum*) and identified several candidate enzymes involved in the downstream biosynthesis of podophyllotoxin: O-methyltransferases (OMT1, OMT2, OMT3, OMT4), CYPs, and a 2-oxopentanedioic acid/Fe(II)-dependent dioxygenase (2-ODD). These enzymes were co-expressed with CYP719A23 in tobacco leaves, and it was found that only *ShOMT3* could catalyze the C-4′ hydroxylation of (−)-pluviatolide to produce (−)-5′-desmethoxy-yatein.

Various enzyme-coding genes (Xu et al., 2024), transcription factors (Nag et al., 2020), and ABC family transporters (Wiese and Stefan, 2019) have regulatory effects on the biosynthesis of PTOX and influence the accumulation of PTOX. *SDH* enzyme gene has been shown to play an essential role in the synthesis pathway of pinoresinol to matairesinol (Arneaud and Porter, 2015). The enzyme proteins encoded by the cytochrome enzyme genes *CYP71BE54* and *CYP82D61* can catalyze the conversion of deoxypodophyllotoxin to 4’-demethylpodophyllotoxin. It further catalyzes the conversion of 4’-demethylpodophyllotoxin to 4’-demethylepipodophyllotoxin (Xu, 2023). The binding sites of transcription factors MYB and WRKY usually exist in the promoter regions of pathway genes such as *CAD*, *PAL*, *PLR*, and *SIRD*. These transcription factors regulate the biosynthesis of PTOX by complementary binding to the sequence elements in the target gene promoter (Kumar et al., 2017). Furthermore, studies have shown that ABC transporters encoded by *PhABC6* and *PhABCIII* genes are positively correlated with PTOX content.

In *S. hexandrum* samples from different provinces in China, PTOX content was sequenced as Ningxia > Gansu > Sichuan > Yunnan (Li et al., 2015). It is concluded that the high altitude areas may be conducive to the high accumulation of PTOX and may also be related to the growth years. Liu et al. (2015) also found environmental factors have obvious effect on secondary metabolites of *S. hexandrum* from different production areas. In Jingyuan of Ningxia Province and Yongdeng of Gansu Province, environmental conditions are appropriate to the production of podophyllotoxin and other lignans. The content of PTOX is significantly different in *S. hexandrum* as the growth location, organ or provenance (Zhao et al., 2023). However, limited studies are available, which obstructs the innovative development and utilization of *S. hexandrum* resources. Clarifying the molecular mechanism of PTOX content difference among different provenances can more effectively play its medicinal role. In this study, *S. hexandrum* samples from different provenances were used as the test material, and metabolomics and transcriptomics techniques were used to explore candidate genes, transcription factors and transporters among the different *S. hexandrum* provenances. The work can provide a new perspective for the causes of the difference in PTOX content of *S. hexandrum* rhizome from different provenances and be helpful for understanding the molecular mechanism of PTOX biosynthesis.

## Materials and methods

### Plant materials

In the year 2022, plant materials were collected from the germplasm resource repository of *S. hexandrum* in Henan University of Science and Technology in China (E112°25′23″, N34°35’46”). This germplasm resource repository was built using the seeds or seedling of *S. hexandrum* collected from different growth locations including mountain Taibai of Mei county in ShaanXi (SX), Bayi town of Nyingchi city in Tibet (XZ), and Yongdeng county of Lanzhou city in Gansu (GS) and other regions. This experiment design is commonly named as the homogenous garden experiment, which ensured the same plant growth environment conditions to study the molecular mechanism of PTOX content difference in different *S. hexandrum* provenance in the view of the molecular. Considered the plant age could influence the contents of phytochemicals, rhizomes of *S. hexandrum* with the same growth age of 5 years from SX, XZ, and GS provenances were selected as test materials. *S. hexandrum* is a perennial rhizome-type plant. The age of the plant is recognized depending on the first emergence time of the seedling generated from the seed sowed in the soil. After sampling, it was rinsed with distilled water, frozen in liquid nitrogen, and then sub-packed appropriately. Finally, these samples were stored in a refrigerator at −80°C for later use.

### Metabolomics analysis

The rhizomes were ground into powder and were accurately weighed 0.1 g, and 500 μL 80% methanol (containing 0.1% formic acid) aqueous solution was added. After vortex oscillation, the supernatant was centrifuged at 4°C for 10 min (15000 rpm), and it was diluted with mass spectrometry-grade water. It was diluted to 53% methanol content and centrifuged again. The supernatant was collected for liquid chromatography-mass spectrometry (LC-MS) analysis (Lone et al., 2023). The quality control sample (QC) was prepared by mixing the supernatant of all test samples in equal volume, and 53% methanol (containing 0.1% formic acid) aqueous solution was used as a blank sample (Blank). Three replicates were set for each sample.

The chromatographic column was the Hypesil Gold column (C18). In the positive ion mode, the mobile phase A is 0.1% formic acid; the mobile phase B is methanol. In the negative ion mode, the mobile phase A was 5 mM ammonium acetate; the mobile phase B was methanol. The gradient elution procedure was 0-1.5 min, 98% A and 2% B; 1.5–12 min, 98% - 0% A and 2% - 100% B; 12–14 min, 0% A and 100% B; 14-14.1 min, 0% - 98% A and 100% - 2% B; 14.1–17 min, 98% A and 2% B. The injection volume was 20 μL, the flow rate was 0.2 mL min^-1^, and the column temperature was 40°C (Xie et al., 2021). The ESI ion source was set to spray voltage 3200 V (positive ion mode) and -3200 V (negative ion mode). The capillary temperature is 320 °C. The sheath gas (nitrogen) flow rate was 40 Arb, and the auxiliary gas flow rate is 10 Arb. The scanning range was 70–1050 m z^-1^ (Yang et al., 2022). Compound Discoverer 3.1 (CD3.1, Thermo Fisher) software was used to screen the retention time, mass-to-charge ratio, and other parameters of the original files detected by mass spectrometry. Peak alignment and peak extraction were performed on different samples by retention time deviation (0.2 min), mass deviation (5 ppm), signal intensity deviation (30%), and additive ions. The peak area was quantified, and the target ions were integrated. Molecular ion peaks and fragment ions predicted the molecular formula. The mzCloud, mzVault and Masslist databases were used for comparison. Blank samples removed background ions, and the quantitative results were normalized. Finally, the qualitative and quantitative results of metabolites can be obtained (He et al., 2021). All differential metabolites were annotated by the KEGG pathway in KEGG data (P ≤ 0.05). The screening conditions for differential metabolites were VIP > 1.0 and FC > 1.5 or FC < 0.667 and P value < 0.05 (Liu et al., 2020).

### Transcriptomics analysis

RNA was extracted using a plant polysaccharide polyphenol total RNA extraction kit. The quality of RNA samples was ensured by detecting integrity, concentration, and purity (1.8 < OD 260/280 < 2.0, RNA concentration > 400 ng μL^-1^, RIN value (RNA integrity number) ≥ 6.5, 28S/18S ≥ 1.0) (Zhang et al., 2022). The mRNA library was constructed using Illumina’s library kit (NEBNext^®^ UltraTM RNA Library Prep Kit) and then sequenced. Three copies of each sample were selected for biological repetition. After sequencing, Cutadapt was used to remove the 3’ end band junction and Reads with an average mass fraction lower than Q20. Trinity software was used to splice Clean Reads to obtain transcripts (Martin-Pizarro et al., 2021).

Transcripts were functionally annotated (E-value ≤ 10^-5^) through the seven databases including Nr (NCBI non-redundant protein sequences), Nt (NCBI nucleotide sequences), Pfam (Protein family), KOG/COG (KOG, euKaryotic Ortholog Groups; COG, Clusters of Orthologous Groups of Proteins), Swiss-prot (a manually annotated and reviewed protein sequence database), KEGG (Kyoto Encyclopedia of Genes and Genomes), and GO (Gene Ontology) (Gao et al., 2024). RSEM software was used to calculate the FPKM value of each gene to analyze gene expression. Differential analysis of gene expression was performed by DESeq (|log2FoldChange| > 1, P-value < 0.05), and KEGG enrichment analysis was performed at P ≤ 0.05 (Guo et al., 2023). Transcription factors and their family information were predicted by Plant TFDB (Plant Transcription Factor Database) (Kumari et al., 2014). Each sample was set to repeat three times.

### Statistical analysis based on multi-omics dada

The correlation analysis (a pearson correlation analysis) of metabolomics and transcriptomics data was carried out to explore the molecular components that regulate PTOX content differences in different provenances. The correlation network diagram was drawn using the visualization software Cytosacpe and Rstudio. The significant value of all statistical differences was set at P < 0.05.

## Results

### Different accumulation metabolites (DAMs) in different *S. hexandrum* provenances

**Figure 1:**
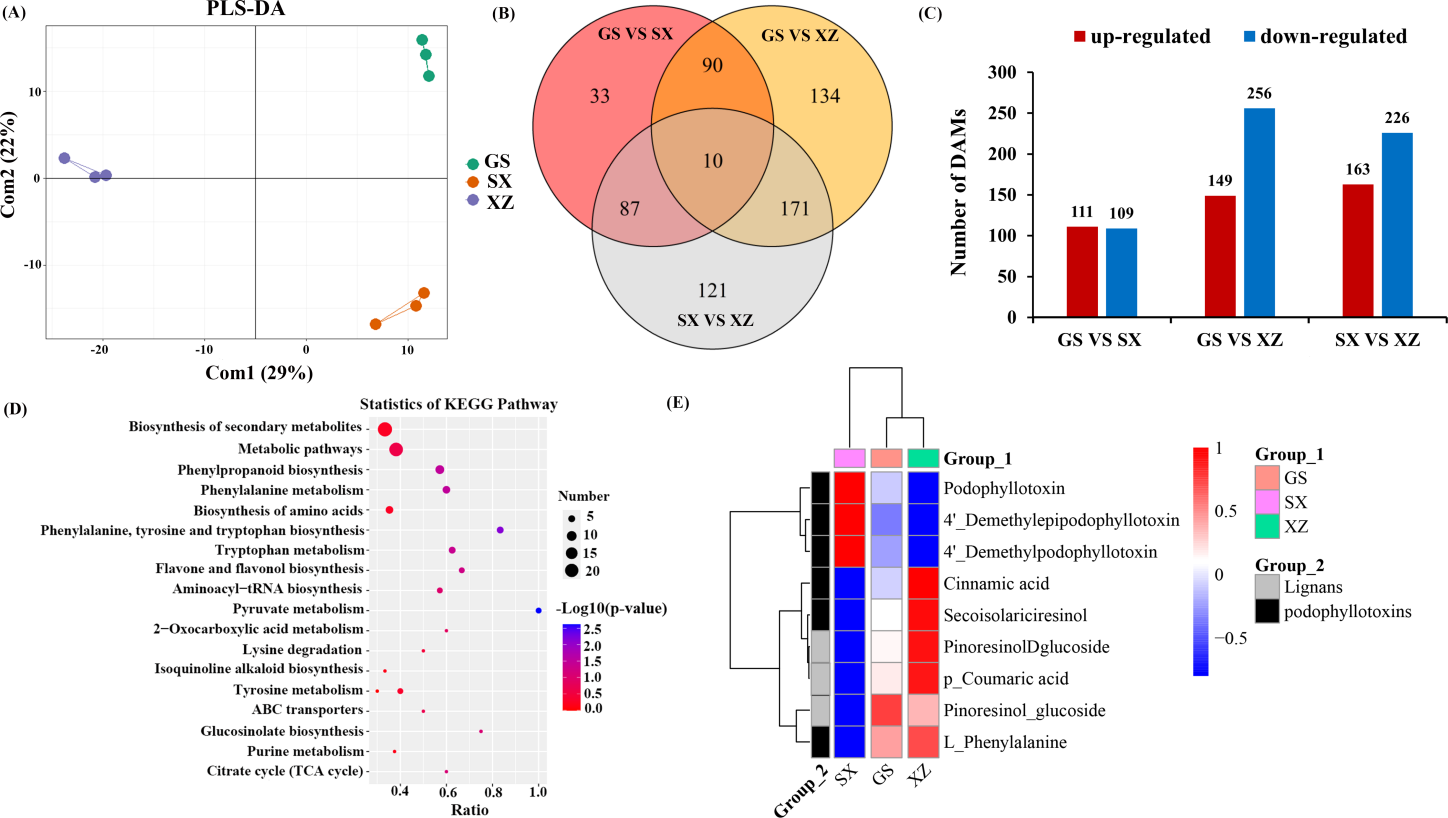
Differential accumulation metabolites (DAMs) analysis related to PTOX biosynthesis. **(A)** PLS-DA analysis of DAMs among groups. The abscissa is the sample’s score on the first principal component (Com1), and the ordinate is the sample’s score on the second principal component (Com2). **(B)** The Venn diagram of GS VS SX, GS VS XZ, and SX VS XZ. **(C)** The number of up-regulated and down-regulated DAMs between GS VS SX, GS VS XZ, and SX VS XZ, respectively. **(D)** KEGG enrichment analysis of DAMs between groups. **(E)** DAMs clustering heat map. The red indicates high expression, and the blue indicates low expression.

This study performed non-targeted metabolomics analysis to identify DAMs among the rhizomes of GS, SX, and XZ provenances. A total of 646 DAMs were identified in three provenances. PLS-DA showed significant aggregation of DAMs between different groups (**Figure 1A**). GS VS SX, GS VS XZ, and SX VS XZ contained 220, 405, and 389 DAMs, respectively. There were 10 common DAMs among the three groups (**Figure 1B**). 423 DAMs were up-regulated, and 591 DAMs were down-regulated. The difference in metabolites between GS and XZ was the largest, and the number of up-regulated DAM was significantly higher than that of the other two comparison groups (**Figure 1C**). KEGG enrichment analysis showed that DAMs among three groups were mainly enriched in the biosynthesis of secondary metabolites, metabolic pathways, phenylpropanoid biosynthesis, phenylalanine metabolism, amino acid biosynthesis, and other pathways (**Figure 1D**).

There were three lignan synthesis-related substances (pinoresinol-glucoside, pinoresinol-dglucoside, p-coumaric acid) and six podophyllotoxin-like substances (L-phenylalanine, cinnamic acid, secoisolariciresinol, 4’-demethylpodophyllotoxin, 4’-demethylepipodophyllotoxin, PTOX) in common DAMs among three groups. Among the six podophyllotoxin-like substances, L-phenylalanine, cinnamic acid and secoisolariciresinol are essential precursors for PTOX biosynthesis. The content of each substance showed significant differences between groups. The contents of three lignan synthesis-related substances and three PTOX precursors were the lowest in SX, the highest in XZ, and the others were the opposite. Most common DAMs were in the middle of GS (**Figure 1E**). These results showed that the expression of three lignan synthesis-related substances and three PTOX precursors were consistent between the groups, with low expression in SX and high expression in XZ. However, the expression of the other three podophyllotoxin-like substances in the group was opposite to the above six substances. The difference in the content of the three precursors and the other three podophyllotoxin-like substances is a critical discovery for different *S. hexandrum* provenances.

### Differentially expressed genes in different *S. hexandrum* provenances

**Figure 2:**
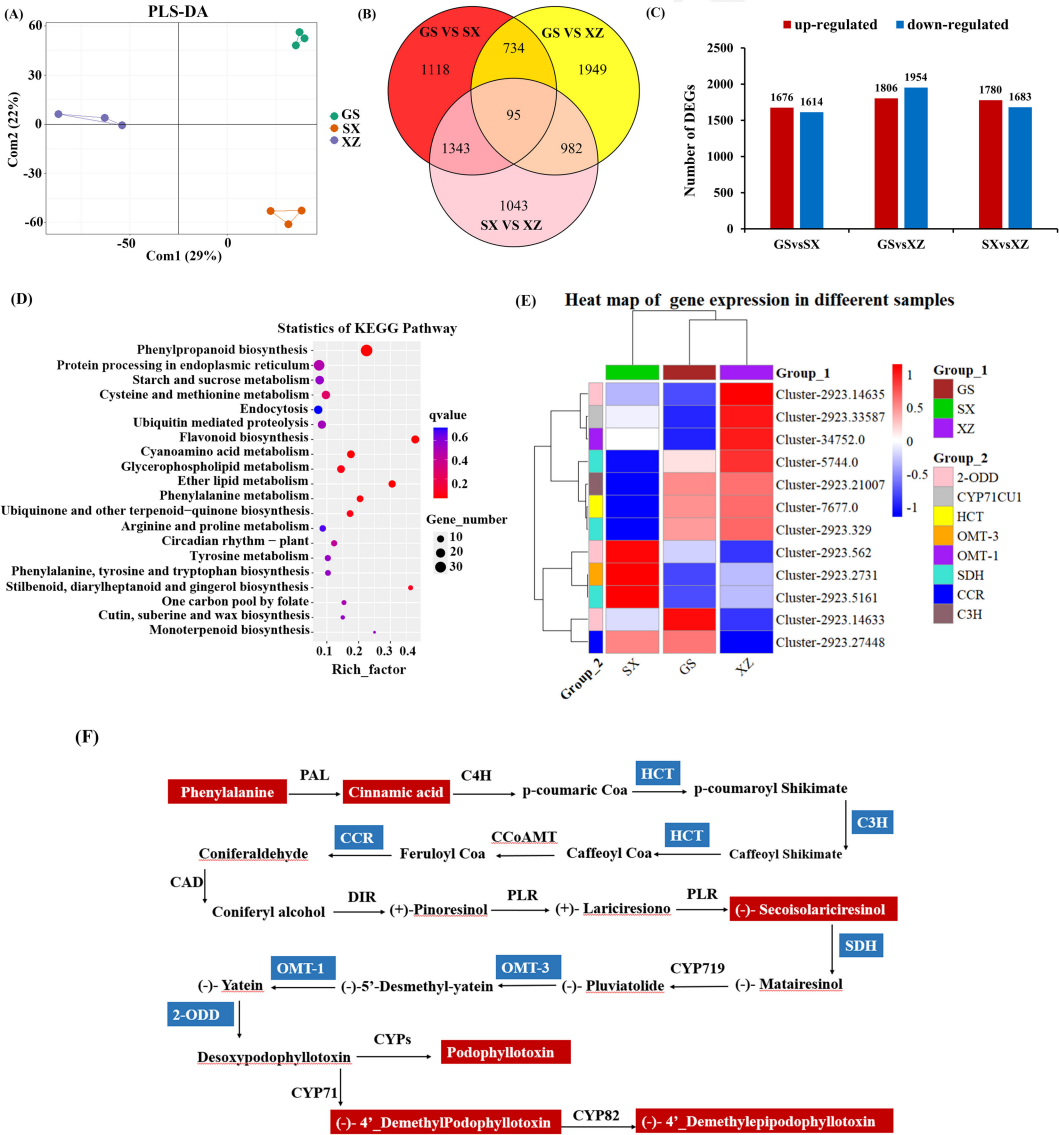
Differentially expressed genes (DEGs) analysis related to PTOX biosynthesis. **(A)** PLS-DA analysis of DEGs among groups. The abscissa is the sample’s score on the first principal component (Com1), and the ordinate is the sample’s score on the second principal component (Com2). **(B)** The Venn diagram of GS VS SX, GS VS XZ, and SX VS XZ. **(C)** The number of up-and down-regulated DEGs between GS VS SX, GS VS XZ, and SX VS XZ, respectively. **(D)** KEGG enrichment analysis of DEGs between groups. **(E)** DEGs clustering heat map. The red indicates high expression, and the blue indicates low expression. **(F)** Schematic diagram of PTOX biosynthesis pathway.

Transcriptome analysis showed that there were 7264 DEGs in the rhizomes of GS, SX, and XZ, and the content of common DEGs was significantly different among groups. The DEGs of different groups were significantly separated (**Figure 2A**). The range of DEGs among groups was 3290-3760. 5262 DEGs were up-regulated, and 5251 DEGs were down-regulated. GS VS SX, GS VS XZ and SX VS XZ contained 3290, 3760 and 3463 DEGs, respectively. 95 DEGs existed in the three groups (**Figures 2B, C**). DEGs analysis results showed that the difference in gene expression between GS and XZ was the largest, including 3760 DEGs, of which 1806 genes were up-regulated, and 1954 genes were down-regulated. KEGG enrichment analysis showed that DEGs were widely distributed in phenylpropanoid biosynthesis, protein processing in the endoplasmic reticulum, starch, and sucrose metabolism (**Figure 2D**). Specially, 12 DEGs were related to PTOX biosynthesis in the 95 common DEGs, and their expression levels had significant differences. The 12 DEGs belongs to 8 kinds of enzyme genes, including *2-ODD* gene (Cluster-2923.562, Cluster-2923.14635, Cluster-2923.14633), *CYP71CU1* gene (Cluster-2923.33587), *HCT* gene (Cluster-7677.0), *OMT-3* gene (Cluster-2923.2731), *OMT-1* gene (Cluster-34752.0), *C3H* gene (Cluster-2923.21007), *SDH* gene (Cluster-2923.5161, Cluster-5744.0, Cluster-2923.329) and *CCR* gene (Cluster-2923.27448) (**Figure 2E**).

Seven DEGs, including Cluster-2923.14635, Cluster-2923.33587, Cluster-34752.0, Cluster-5744.0, Cluster-2923. 21007, Cluster-7677.0 and Cluster-2923.329, were lowly expressed (down-regulated) in SX and highly expressed (up-regulated) in XZ, in contrast with the remaining. These seven DEGs belong to seven enzyme genes (*2-ODD*, *CYP71CU1*, *OMT-1*, *SDH*, *C3H*, *HCT*, *SDH*), respectively. The expression of the seven enzyme genes significantly differed between the groups, indicating that they may play an essential role in regulating PTOX biosynthesis. Metabolomics and transcriptomics analysis identified the differences in metabolites and gene expression for different *S. hexandrum* provenances: 6 DAMs and 12 DEGs directly related to PTOX biosynthesis. Pearson correlation analysis was also performed between the identified 7264 DEGs and the above six key DAMs, and a total of 1899 genes significantly associated with DAMs were screened (Pearson correlation coefficient ≥ 0.80 or ≤ − 0.80, P < 0.05). The results showed that four of the 12 DEGs were highly correlated with DAMs, including two 2-ODD enzyme genes (Cluster-2923.14635, Cluster-2923.14633), one C3H enzyme gene (Cluster-2923.21007), and one SDH enzyme gene (Cluster-2923.329).

Podophyllotoxin biosynthesis originates from pinoresinol, a common precursor of many lignans. The biosynthesis of podophyllotoxin can be divided into two major stages (Danaeipour et al., 2023; Palaniyandi and Jun, 2020): (1) the phenylpropanoid pathway, where pinoresinol is synthesized through a series of enzymatic reactions, including deamination, hydroxylation, methylation, acetylation, and redox modifications of phenylalanine. Related enzymes and proteins have been found. For example, phenylalanine ammonia-lyase (PAL), p-hydroxycinnamoyl-CoA (HCT), cinnamoyl-CoA reductase (CCR), cinnamate 4-hydroxylase (C4H), cinnamyl alcohol dehydrogenase (CAD) and DIR proteins are involved in this stage (Palaniyandi and Jun, 2020); and (2) the podophyllotoxin biosynthetic pathway, in which pinoresinol undergoes further transformations to produce podophyllotoxin and its derivatives by a series of enzyme catalytic role such as DIR, PLR and SDH, accumulating in plant cells and tissues. These compounds are predominantly stored as chemically modified glycosides (**Figure 2F**). It was therefore speculated that the significant differences in these metabolites were caused by the differential expression of genes.

### Key gene modules by WGCNA analysis based on multi-omics data

**Figure 3:**
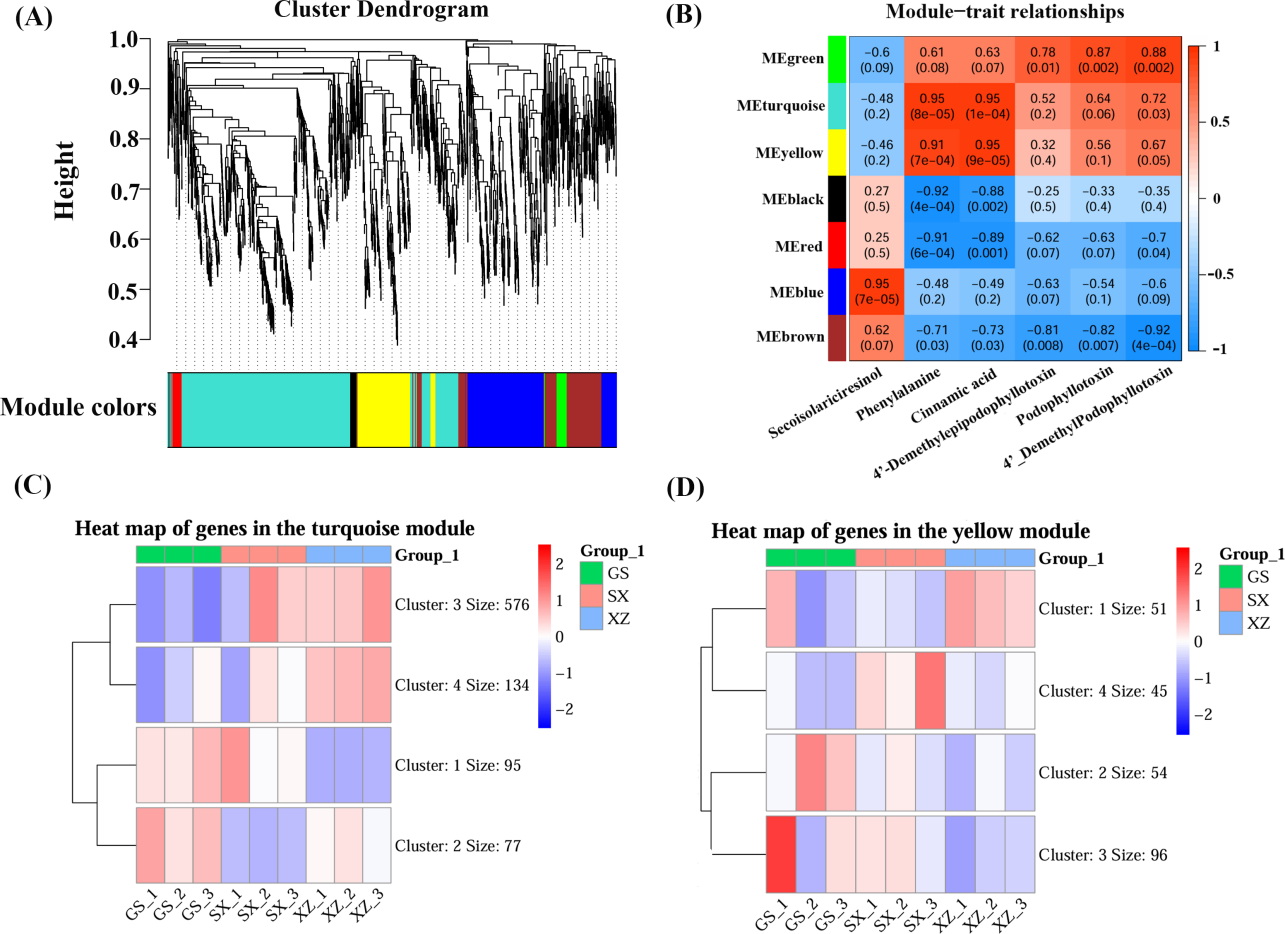
Weighted gene co-expression network analysis (WGCNA) of DEGs and gene expression patterns in different modules. **(A)** Hierarchical clustering tree. **(B)** Correlation between gene modules and DAMs. **(C)** Heat map of genes in the turquoise module. **(D)** Heat map of genes in the yellow module.

The relationship between important substances and related genes was determined by weighted gene co-expression network analysis (WGCNA) of 1899 DEGs and 6 DAMs. The hierarchical clustering tree showed that 1899 genes were clustered into seven modules. The seven modules were marked with different colors: green module, turquoise module, yellow module, black module, red module, blue module, and brown module (**Figure 3A**). Modules represent highly correlated gene clusters, and they are co-expressed in the same module. The turquoise and the yellow module positively correlated with phenylalanine and cinnamic acid (r > 0.9, P < 0.05). Two 2-ODD enzyme genes (Cluster-2923.14635, Cluster-2923.14633), C3H enzyme gene (Cluster-2923.21007), and SDH enzyme gene (Cluster-2923.329) were clustered in the yellow module. The results demonstrated that the turquoise and yellow modules are significantly associated with PTOX contents, and the genes expression involved in the modules can influence the PTOX biosynthesis and be used for subsequent analysis (**Figure 3B**). In **Figure 3B**, each row represents a module, and the color of each module is displayed on the left side. Each column represents a kind of metabolite. The value indicates the correlation coefficient between the module and the metabolite in the table cell at the intersection of the row and the column. The closer the value is to 1, the stronger the positive correlation; the closer to -1, the stronger the negative correlation. The number in brackets represents the significance level viz. P value. The smaller the P value, the stronger the significance.

The FPKM value of the genes in the module was used to draw the gene expression clustering heat map to further determine the expression pattern for the genes in turquoise and yellow modules. The red indicates high expression, and the blue indicates low expression (**Figures 3C, D**). The heat map results showed that the genes in the turquoise and yellow modules were clustered into four clusters (Clusters 1-4). There were 882 genes in the turquoise module, and 95 and 77 genes in Cluster1 and Cluster2 were highly expressed in GS. Cluster 3 and Cluster 4 contained 576 and 134 genes, highly expressed in XZ (**Figure 3C**). The results showed that most of genes in the turquoise highly expressed in XZ compared with other *S. hexandrum* samples, which might be a reason why PTOX content is much higher in *S. hexandrum* from Tibet (XZ). The yellow module contains 246 genes, and the number of genes in the four clusters (Clusters 1-4) is 51, 54, 96, and 45, respectively. Cluster1 is highly expressed in XZ, while Cluster2 is highly expressed in GS. Cluster3 was highly expressed in GS and SX, and Cluster4 was highly expressed in SX (**Figure 3D**). Although the turquoise and yellow modules were significantly related to the PTOX and its related substances, their gene expression patterns were significantly different.

Obviously, the turquoise and yellow modules played different roles in the PTOX biosynthesis process in terms of time and space. In general, DEGs that high expression accounts for a relatively large proportion in the turquoise module, which indicated that genes in the turquoise module play a great role for the formation and accumulation of high-content PTOX. These differentially expressed genes were transcribed to synthesize mRNAs. mRNAs translated amino acids into polypeptides, which were ultimately processed into various enzymes (proteins) that can catalyze the biosynthesis of PTOX. Transcription and translation of genes determines the types, quantities and sequence of amino acids in polypeptides, and the catalytic activities of enzymes are different. This may directly or indirectly affect the metabolism of PTOX, and thus showed content differences in different *S. hexandrum* provenances. Of course, this can also lead to differences in the composition, structure and proportion of secondary metabolites.

### Gene function analysis based on KEGG enrichment analysis

**Figure 4:**
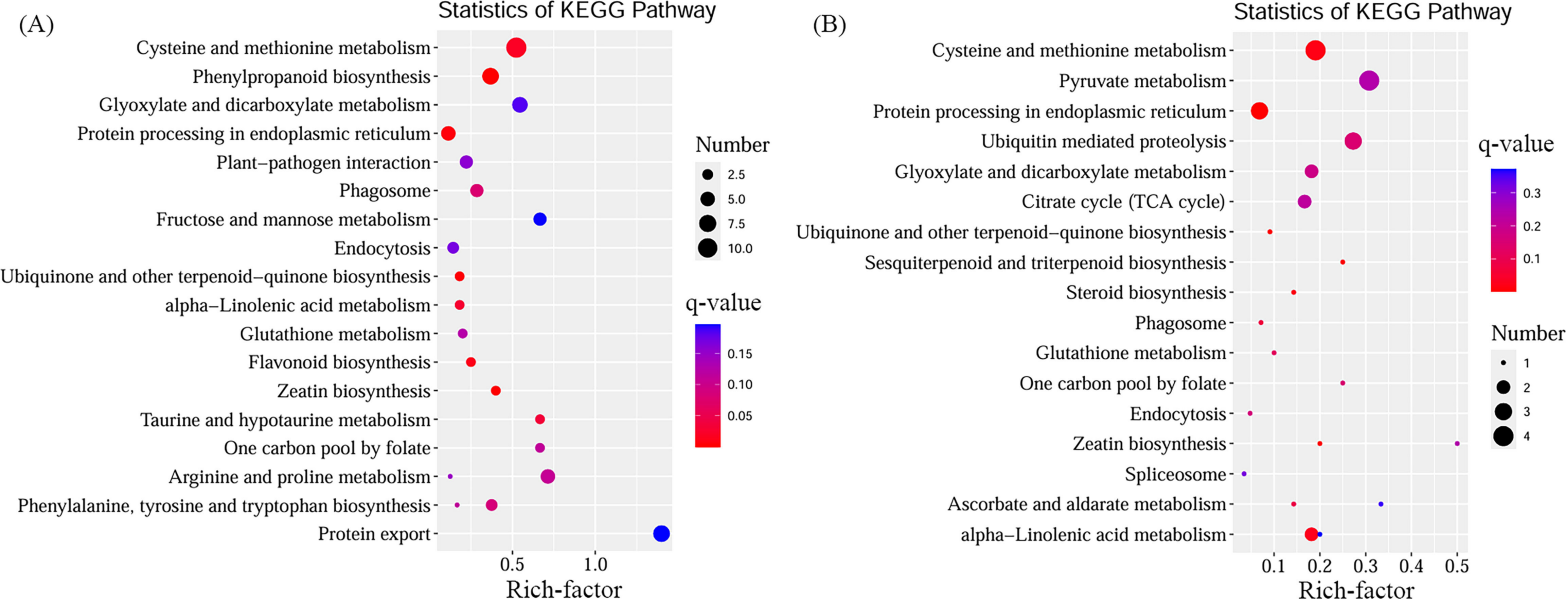
Enrichment analysis of genes in turquoise module **(A)** and yellow module **(B)**.

To determine the relationship between genes and metabolites, the function of genes in the module was first determined by KEGG enrichment analysis. KEGG enrichment analysis showed that cysteine and methionine metabolism, phenylpropanoid biosynthesis, and protein synthesis were the most significant pathways in the turquoise module (**Figure 4A**). The genes in the yellow module were significantly enriched in pyruvate metabolism, cysteine, and methionine metabolic pathways (**Figure 4B**). The only first 20 significantly enriched pathways were visually displayed in these two modules. The yellow module contained two 2-ODD enzyme genes (Cluster-2923.14635, Cluster-2923.14633), one C3H enzyme gene (Cluster-2923.21007), and a SDH enzyme gene (Cluster-2923.329). The gene expression patterns of the two modules were significantly different. The related genes were significantly enriched in cysteine and methionine metabolism, phenylpropanoid biosynthesis, protein synthesis, and pyruvate metabolic pathways.

### Excavation of molecular elements related to podophyllotoxin contents difference

**Figure 5:**
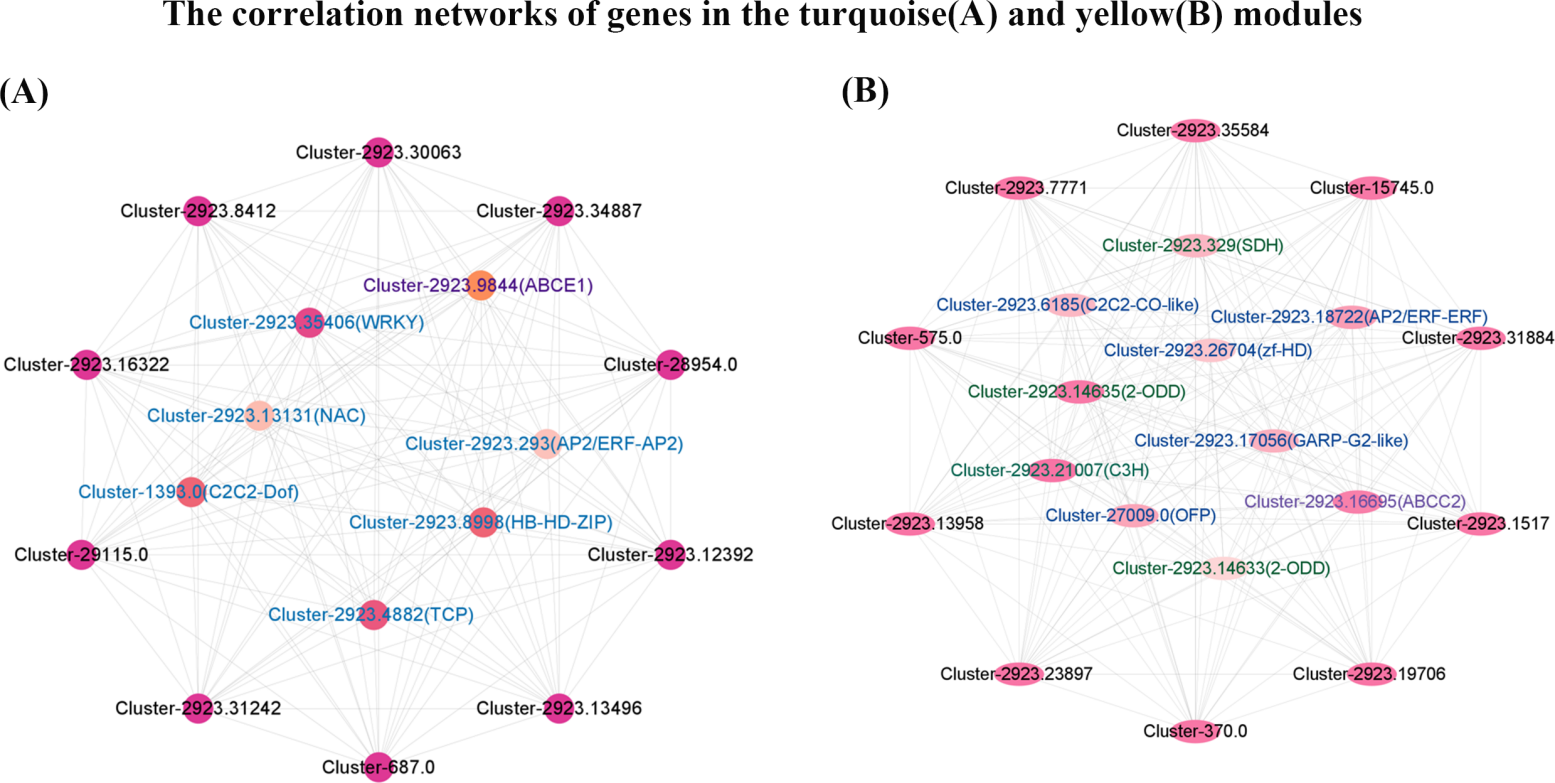
Turquoise module **(A)** and yellow module **(B)** related gene network diagram. The color of the circle represents the degree value. The deeper the color, the higher the degree value. The outer ring represents genes with high degree values, and the inner ring represents structural genes (green fonts), transcription factors (blue fonts), and transporters (purple fonts).

The expression and function of genes in essential modules have been clarified. However, the critical molecular elements in the module are still unclear. The connectivity between any genes in the module was evaluated by calculating the degree value (threshold > 0.4, weight > 0.4). The correlation network diagram of the turquoise and yellow modules was further constructed to determine the co-expression relationship between any genes in the module and screen the key molecular elements. The turquoise module included six transcription factors (WRKY, AP2/ERF-AP2, NAC, C2C2-Dof, TCP, HB-HD-ZIP) and one transporter (ABCE1) (threshold > 0.4, weight > 0.4). The correlation network diagram was drawn using the above 7 genes and the other 10 genes with the highest connectivity in the module. Transcription factors and transporters correlated highly with the top 10 genes with the highest connectivity (**Figure 5A**). Transcription factors were also significantly correlated with each other. Among the six selected transcription factors, WRKY had the highest connectivity with other genes in the module. Four PTOX synthesis structural genes (two 2-ODD enzyme genes, Cluster-2923.14635, Cluster-2923.14633; one C3H enzyme gene, Cluster-2923.21007; one SDH enzyme gene, Cluster-2923.329), five transcription factors (C2C2-Co-like, AP2/ERF-ERF, zf - HD, GARP-G2-like, OFP) and one transporter (ABCC2) were screened from the yellow module (threshold > 0.4, weight > 0.4). The top 10 genes with the highest connectivity in the yellow module were used to draw a correlation network diagram with the above-selected genes and transcription factors (**Figure 5B**). Structural genes, transcription factors, transporters, and genes with the highest connectivity were significantly correlated. Among the five selected transcription factors, the AP2/ERF-ERF transcription factor has the highest connectivity with other genes in the module.

Obviously, the structural genes *2-ODD*, *SDH*, and *C3H* may play a vital role in the biosynthesis of PTOX. Therefore, this study speculates that *2-ODD*, *SDH*, and *C3H* are the essential genes regulating the difference of PTOX content in different *S. hexandrum* provenances. WRKY and AP2/ERF-ERF are key transcription factors, and ABCE1 and ABCC2 are the main proteins for PTOX transport.

## Discussion

Generally, DEG was positively correlated with DAM, which means that the more DEGs, and the more DAMs (Danaeipour et al., 2023). In present study, the contents of three lignans (4’-demethylpodophyllotoxin, 4’-demethylepipodophyllotoxin, PTOX) were significantly different among groups. The transcription level of the structural gene *CCR* is positively correlated with the PTOX content in the PTOX biosynthesis pathway (Kumar et al., 2016). The *CCR* gene identified in this study was highly expressed in SX and lowly expressed in XZ. In addition, these three metabolites had the highest content in SX and the lowest content in XZ, which is consistent with results from previous studies. Therefore, the expression level of the *CCR* gene is speculated as one reason for the PTOX content difference among groups. Many precursor substances were catalyzed to form PTOX by a series of enzymes. L-phenylalanine, cinnamic acid and secoisolariciresinol are precursors in the synthesis pathway of PTOX. However, their contents change trend was opposite to PTOX, which was consistent with the three lignans (pinoresinol-glucoside, pinoresinol-dglucoside, p-coumaric acid), viz. the lowest PTOX content in SX and the highest content in XZ. Transcriptome analysis showed structural genes *2-ODD* (Cluster-2923.14635), *CYP71CU1* (Cluster-2923.33587), *OMT-1* (Cluster-34752.0), *SDH* (Cluster-5744.0), *C3H* (Cluster-2923.21007), *HCT* (Cluster-7677.0), *SDH* (Cluster-2923.329) were lowly expressed in SX and highly expressed in XZ. The expression of these genes promoted the accumulation of PTOX synthesis precursors. In *S. hexandrum*, the total lignans contents is a constant value. The increase of other lignans content will lead to the decrease of PTOX content. Moreover, there is an interaction among genes. A network will be formed among them to jointly regulate the synthesis of secondary metabolites. The high expression of these genes inhibits the expression of synthase genes and promotes the expression of decomposition enzyme genes in the PTOX biosynthesis pathway. Therefore, the content of PTOX decreased. The high expression of the above seven enzyme genes may provide sufficient precursors for the biosynthesis of PTOX. Many structural genes produce precursors, such as p-coumaric acid and phenylalanine. However, these intermediates can synthesize PTOX and provide substrates for the biosynthesis of other lignans. In addition, precursors are also transferred into other plant organs. This may be a reason why the changing trend of PTOX synthesis precursor content is inconsistent with PTOX content, nevertheless the changing trend of PTOX content is the same with lignans.

The functions of genes in the turquoise and yellow modules are different, so the expression patterns of genes are also different. The genes in the turquoise module are mainly related to phenylpropanoid biosynthesis and protein synthesis. Phenylpropanoid biosynthesis is the initial step of PTOX biosynthesis. The precursor of lignans, coniferyl alcohol, is primarily produced through the phenylpropanoid pathway (Li et al., 2018). The turquoise module is significantly associated with the PTOX synthesis pathway, so the genes in the turquoise module are significantly enriched in the phenylpropanoid biosynthesis pathway. The genes in the yellow module were significantly enriched in pyruvate, cysteine and methionine metabolism. Transcriptome analysis of *S. hexandrum* rhizomes from different regions has identified that DEGs related to PTOX synthesis are mainly enriched in the carbon metabolic pathway (Nag et al., 2020). Although the carbon metabolic pathway in the yellow module is not significantly enriched, pyruvate metabolism is an integral part of carbon metabolism. Therefore, it is also critical in the metabolic pathway of plants, which also confirms the results of the present study.

*S. hexandrum* is an endangered anti-cancer medicinal plant, primarily grown in alpine meadow or forest margins at the probable altitude range of 2400–4500 m. The content of PTOX and the expression of related genes are highly susceptible due to environmental conditions. For instance, temperature, illumination, moisture and soil (pH, macronutrients, micronutrients, nutrient availability) are external factors affecting PTOX content and gene expression. The content of PTOX and the expression of related genes in *S. hexandrum* were significantly different at different temperatures. The overexpression of growth-related genes and transcription factor at 15°C resulted in a significant accumulation of PTOX. However, overexpression of genes and transcription factor related to stress tolerance at 25°C resulted in a decrease in PTOX content (Kumari et al., 2014). Compared with other wavelengths of light, red light is most conducive to forming PTOX (Yousefzadi et al., 2012). Wound and UV irradiation can also stimulate the overexpression of critical genes such as *PLR* and *SDH* in PTOX biosynthesis pathway (Wankhede et al., 2013; Yang et al., 2016). The PTOX content of plants grown at high altitudes is about two times higher than that of plants grown at low altitudes (Alam and Naik, 2009). In addition, there are significant differences in PTOX content among the wild *P. hexandrum* (synonym for *S. hexandrum*) populations from different geographical regions (Alam et al., 2009). Obviously, PTOX content is related to a lot of environmental factors. The essential reason may be that these environmental factors regulate the expression of genes related to PTOX biosynthesis. In this study, *2-ODD*, *SDH*, and *C3H* enzyme genes were used for WGCNA analysis. Three genes were clustered into the yellow module, which is highly correlated with the target metabolite PTOX. They not only have a high degree of connectivity in the network diagram but also are highly correlated with transcription factors and transporters. Therefore, it is speculated that *2-ODD*, *SDH*, and *C3H* are essential candidate genes for regulating PTOX content in different provenances.

Transcription factor can regulate the expression of genes by recognizing the sequences in the promoter region and play an important role in regulating plant secondary metabolite biosynthesis (Liao et al., 2008). For example, in the *Podophyllum* species (*S. hexandrum* and *Podophyllum peltatum*), it is found that the binding sites of WRKY transcription factors mostly exist in the promoter region of PTOX synthesis genes (Kumar et al., 2017). AP2/ERF transcription factors have been shown to regulate the synthesis of phenylpropane and lignans (Yang et al., 2012). Furthermore, the correlation network diagram proves that the WRKY in the turquoise module and AP2/ERF-ERF in the yellow module have higher degree values. AP2/ERF-ERF is highly correlated with structural genes and transporters. Therefore, it is speculated that WRKY and AP2/ERF-ERF are key transcription factors in the regulation of PTOX synthesis pathway (Kumar et al., 2017). AP2/ERF-AP2, NAC, C2C2-Dof, TCP, HB-HD-ZIP, and WRKY were co-expressed in the turquoise module. Transcription factors C2C2-Co-like, zf-HD, GARP-G2-like, and OFP were highly correlated with AP2/ERF-ERF in the yellow module. Previous studies have shown that NAC, C2C2-Dof, TCP, and MYB transcription factor families regulate the biosynthesis of plant secondary metabolites (Yang et al., 2012). NAC is a main regulator of plants under drought stress and can help to enhance the tolerance of maize and rice to stress (Wang et al., 2021). These transcription factors can co-regulate plant growth and development (Danaeipour et al., 2023). It has not been reported that regulatory effect of NAC, TCP and C2C2-DOF in PTOX biosynthesis pathway. However, they are essential in plant growth and development and co-expressed with some common transcription factors such as WRKY, AP2/ERF-ERF. Therefore, this study speculated that NAC, TCP and C2C2-DOF also play a role in PTOX biosynthesis.

The formation of natural products is achieved through such a network of “transcription factors – structural genes – enzymes – biosynthesis – metabolites” in plants. Transcription factors play the role of the “master switch” or “conductor” in this network. They determine the “on/off” and “flow rate” of the entire pathway by regulating the expression of structural genes. The mechanism by which transcription factors regulate the biosynthesis of secondary metabolites is an extremely complex and delicate multi-level network. For instance, transcription factors precisely control the spatio-temporal expression of multiple structural genes by directly regulating specific sequences of target gene promoters or by synergistic regulation through forming a complexes with other transcription factors or proteins, thereby determining the accumulation amount, accumulation site and accumulation time of the compound. Transport protein also plays a key role in the synthesis, transport and regulation of plant secondary metabolites, mainly influencing the distribution and accumulation of metabolites through transmembrane transport. Secondary metabolites, such as lignans, alkaloids and flavonoids, are synthesized in cells, and need to be transported to outside of the cell or specific organelles such as vacuoles, mitochondria, endoplasmic reticulum or different tissues via transport proteins. PTOX is synthesized and translocated in the cytosol and stored in the form of glucosides (Meng et al., 2021). Most glycoside derivatives are stored in vacuoles. The ABCC family is related to the vacuolar chelation of glucosides and the transport of PTOX (Nag et al., 2020). In this study, we identified two target transcriptional proteins ABCCE1 and ABCC2 that cause differences in the content of PTOX. As members of the ABCC family, ABCCE1 and ABCC2 are highly correlated with transcription factors and structural genes in the correlation network diagram. Therefore, these two proteins may be the main transporters that regulate the translocation and storage of PTOX in the rhizome of *S. hexandrum*.

## Conclusion

In this study, non-targeted metabolomics and transcriptomics analysis were performed on the rhizomes of different *S. hexandrum* provenances from the same germplasm resource nursery. The results unambiguously identified 6 DAMs and 12 DEGs related to PTOX synthesis. DAMs content and DEGs expression showed significant differences existed among different *S. hexandrum* provenances. WGCNA analysis showed that there was a strong positive correlation between 6 DAMs and 12 DEGs in PTOX synthesis pathway. The genes of *2-ODD*, *SDH*, and *C3H* are candidate genes that regulate the difference of PTOX content in different provenances. WRKY and AP2/ERF-ERF are considered to be the key transcription factors involved in the regulation of PTOX biosynthesis. ABCE1 and ABCC2 are the primary transporters related to PTOX synthesis. The present results excavated some excellent genes for explaining the reasons of PTOX content difference in SX, XZ, and GS from molecular perspective. Meanwhile, it provides a theoretical basis for the exploration of PTOX biosynthesis mechanism.

## Data Availability

The original contributions presented in the study are included in the article/supplementary material. Further inquiries can be directed to the corresponding author.
